# Supplementary material for: Chronotype and trait self-control as unique predictors of sleep quality in Chinese adults: The mediating effects of sleep hygiene habits and bedtime media use
Source: PLoS One. 2022 Apr 15;17(4):e0266874. doi: 10.1371/journal.pone.0266874 (PMC9012385; doi:10.1371/journal.pone.0266874)
Supplement: S1 Table — (PDF) [file pone.0266874.s001.pdf]

Table S1. Results from moderated mediation model

	M1: SH1		M2: SH2		M3: SH3		M4: SH4		M5: Bedtime media use		Y: Sleep quality	
Predictors	*b (SE)*	*p*	*b (SE)*	*p*	*b (SE))*	*p*	*b (SE)*	*p*	*b (SE)*	*p*	*b (SE)*	*p*
Constant	.02 (.14)	.873	.04 (.13)	.770	-.09 (.10)	.381	.19 (.15)	.218	.06 (.18)	.737	4.26 (.10)	.000
Age D1	.11 (.13)	.402	-.07 (.11)	.562	.01 (.09)	.927	.00 (.13)	.982	.16 (.16)	.314	.23 (.06)	.887
Age D2	.59 (.22)	.008	.31 (.20)	.118	-.04 (.16)	.804	-.21 (.23)	.368	-.30 (.28)	.288	.02 (.01)	.628
Gender	-.30 (.14)	.034	-.15 (.12)	.227	.08 (.10)	.425	-.18 (.15)	.217	-.17 (.18)	.330	.01 (.02)	.762
Marital status	.21 (.13)	.094	.17 (.11)	.133	.06 (.09)	.545	-.03 (.13)	.821	.08 (.16)	.606	-.01 (.08)	.550
Trait self-control	.17 (.09)	.070	.16 (.08)	.052	.16 (.07)	.022	.23 (.10)	.019	-.19 (.12)	.097	-.07 (.15)	.000
Chronotype (Morningness)	.05 (.02)	.003	.07 (.01)	.000	.00 (.01)	.737	.03 (.02)	.123	-.08 (.02)	.000	.03 (.10)	.152
Chronotype × Trait self-control	.06 (.02)	.021	.05 (.02)	.030	-.01 (.02)	.591	-.01 (.03)	.765	-.04 (.03)	.242	-.05 (.08)	.506
M1: SH1											.29 (.05)	.000
M2: SH2											.04 (.06)	.466
M3: SH3											.08 (.07)	.226
M4: SH4											.19 (.05)	.000
M5: Bedtime media use											-.10 (.04)	.011
Chronotype × M1											-.04 (.02)	.016
Chronotype × M2											.01 (.02)	.603
Chronotype × M3											-.03 (.02)	.152
Chronotype × M4											-.01 (.01)	.511
Chronotype × M5											-.02 (.01)	.078
	R^2 = .19		R^2 = .21		R^2 = .04		R^2 = .05		R^2 = .12		R^2 = .52	

Note. Age D1 was coded as 0 for 21-30 years and 1 for 31-50 years, whereas age D2 was coded as 0 for 21-30 years and 1 for 50 years or above. Gender was coded 0 for men and 1 for women. Marital status was coded as 0 for single, divorced or widowed and 1 for married. SH1 = pre-sleep stress management, SH2 = sleep timing regularity, SH3 = avoiding sleep-disrupting food or activities, SH4 = keeping a restful sleep environment.
