# Supplementary figures and images for: Chronotype and trait self-control as unique predictors of sleep quality in Chinese adults: The mediating effects of sleep hygiene habits and bedtime media use
Source: PLoS One. 2022 Apr 15;17(4):e0266874. doi: 10.1371/journal.pone.0266874 (PMC9012385; doi:10.1371/journal.pone.0266874)

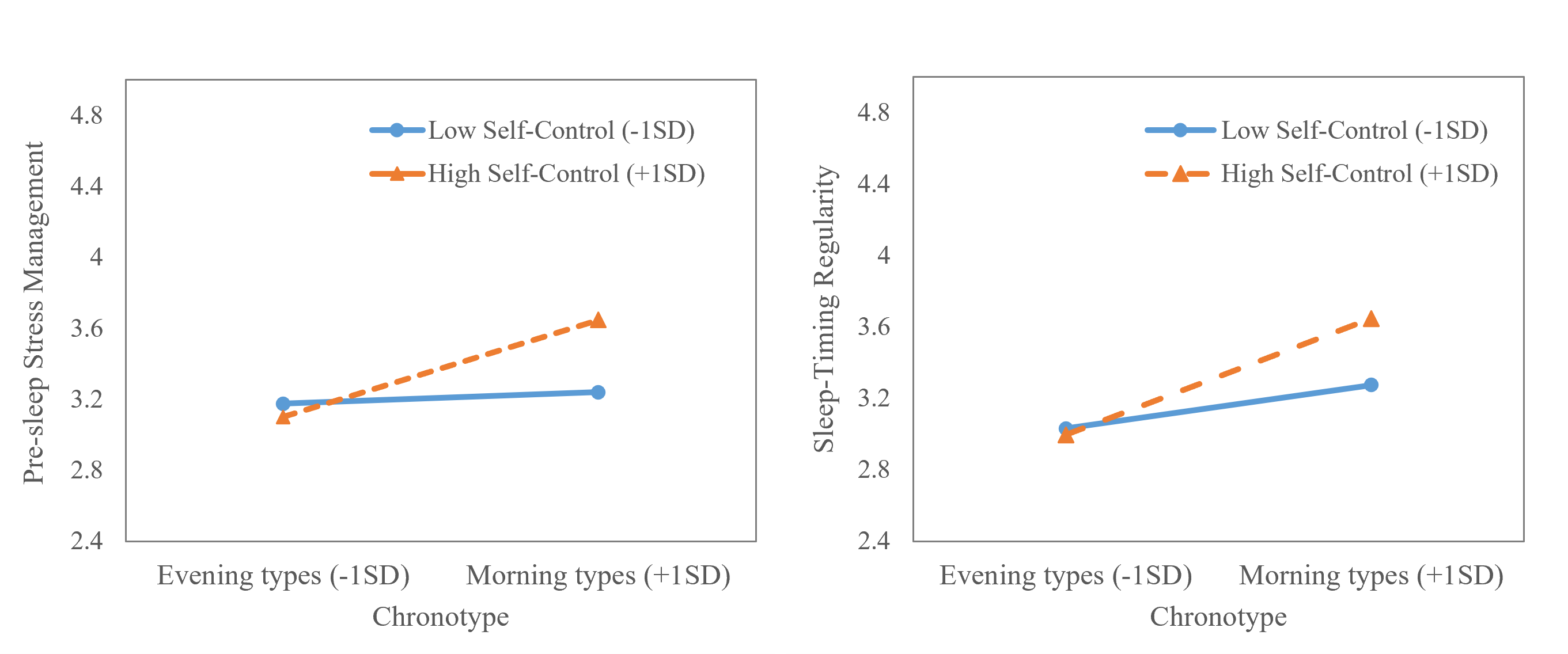

Supplement: S1 Fig — (TIF) [file pone.0266874.s002.tif]

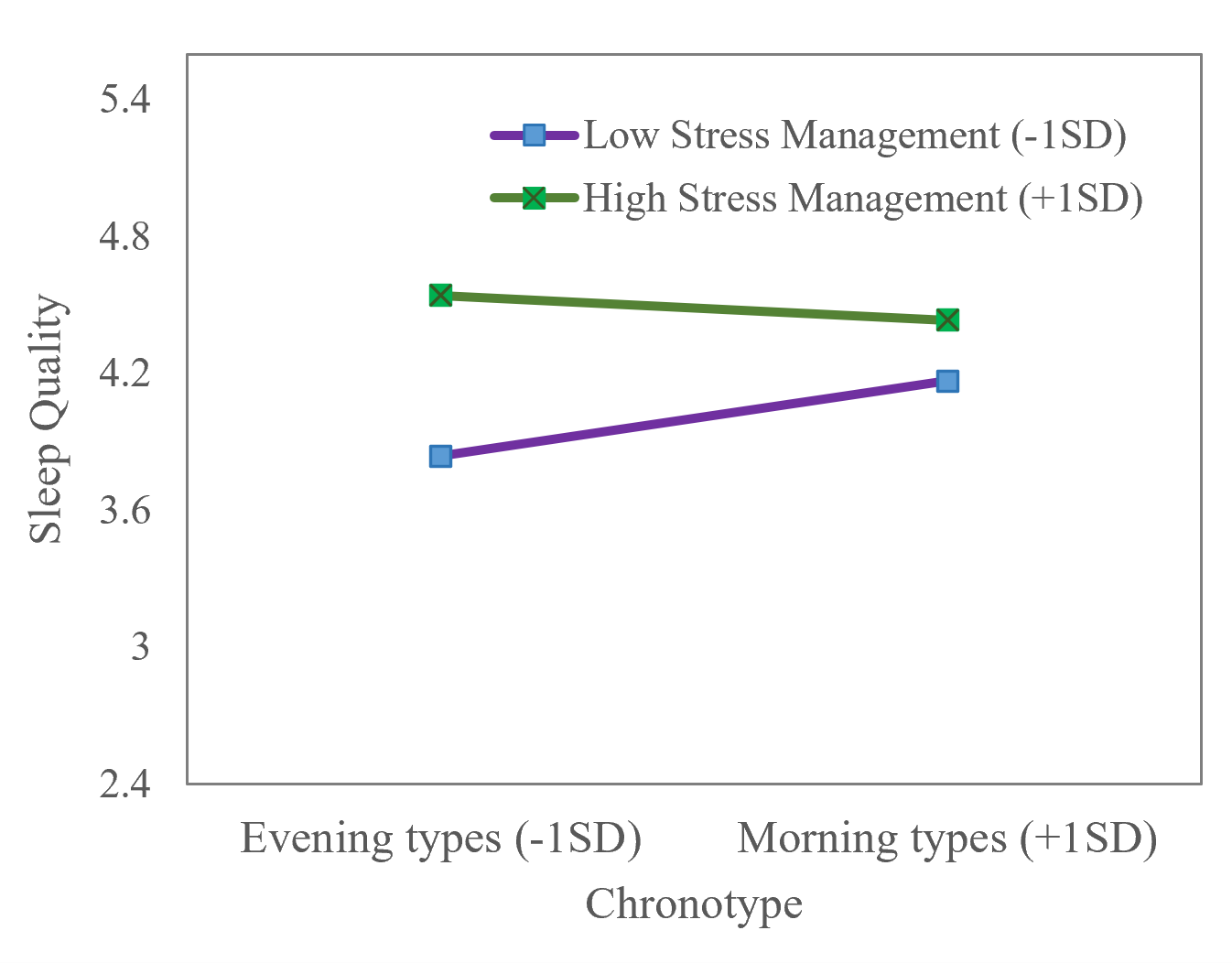

Supplement: S2 Fig — (TIF) [file pone.0266874.s003.tif]
