# Supplementary material for: Chronotype and trait self-control as unique predictors of sleep quality in Chinese adults: The mediating effects of sleep hygiene habits and bedtime media use
Source: PLoS One. 2022 Apr 15;17(4):e0266874. doi: 10.1371/journal.pone.0266874 (PMC9012385; doi:10.1371/journal.pone.0266874)
Supplement: Attachment — Submitted filename: PLOSEOne review_check.docx [file pone.0266874.s004.docx]

**Chronotype and trait self-control** **distinctively predicted subjective sleep quality via sleep hygiene behaviors and bedtime media use**

The authors analyze the influence of chronotype and differences in self-control on the subjective assessment of sleep quality. They assume that the relationship between chronotype, self-control, and sleep quality is mediated by sleep hygiene practices and the bedtime media use.

Below, I make comments and remarks on the following parts of the manuscript.

**Abstract:**

1) The statement (p. 2) "Chronotype and trait self-control positively predicted sleep quality" is imprecise and its correct interpretation requires the knowledge that the higher the score on the chronotype measuring scale, the more morning type the person is. The wording "late chronotype" is likewise imprecise.

**Introduction:**

1) It is not clear to me why the authors start from the self-control strength model and then refer to this model several times since they analyze individual differences in self-control? After all, they do not analyze self-control as a state, but as a trait.
2) The authors wrote that „their study focuses on two individual differences that determine self-regulation abilities - i.e. chronotype and trait self- control ” (p.3). This sentence requires clarification, in what sense does the chronotype determine the ability to self-regulate? The authors mean the general ability to self-regulate, or the ability to self-regulate sleep behavior?
3) Again, the authors describe the self-control strength model and then state that the self-control trait is a stable individual difference (p.3). In the self-control strength model, intra-individual differences in self-control are analyzed, not differences between individuals.
4) The authors repeated that "another characteristic that determines self -regulation abilities and contributes to sleep quality is chronotype." (p.5) Again, are the authors concerned with general self-regulation abilities or just regulation of sleep behavior?
5) In the part describing the relationship between the chronotype and sleep hygiene and sleep quality, the authors mention the relationship between eveningness and procrastination, often confirmed in research (p. 6). However, the authors do not mention that the research also indicates a relationship between chronotype and self-control trait. Thus on the basis of the research conducted so far, the assumption made by the authors about the independent effect of self-control and chronotype seems unjustified. While it is still unclear about the direction of the relationship between self-control and chronotype, it seems obvious that the two features are related. It also means that the model of analysis adopted by the authors in the following part is not justified and raises significant doubts.

**Statistical analysis:**

1) Why do the authors provide age ranges, but mean value, standard deviation, and age range are missing? Why do the authors divide age into two categories under 40 and over 41 instead of including age as a continuous variable? Age is an important factor influencing the chronotype and quality of sleep. With this approach to this variable, the relationship between age and chronotype and sleep quality is impossible to capture.
2) The results of the correlation analysis confirm that self-control and chronotype are significantly (p <.001) related. In my opinion, this relationship does not allow these variables to be included in the analysis as two distinctive independent variables.
3) Further analyzes confirm that the effect of self-control on sleep quality is bigger than that of the chronotype and that it is both direct and indirect.

**Discussion:**

1) The authors wrote: :” In accordance with the self-control strength model, the abilities to downregulate stress and emotion (e.g. self-regulatory abilities) underlie the differences in sleep quality between morning and evening types and between people high vs. low in trait self control”. (p.20) - I do not understand in what sense it is consistent with the self-control strength model? In addition, it is about abilities or some habitual behaviors? Sleep hygiene is associated with certain healthy habits, not abilities.
2) The discussion does not explain the lack of influence on sleep quality in the studied group of such sleep hygiene behaviors as sleep timing regularity and avoiding sleep-disrupting food or activities. Why did these behaviors, known to positively affect sleep quality, not have such an impact on sleep quality in the study group?
3) Pointing to the limitations of the conducted study, the authors emphasize that sleep habits should be measured in a different way in the future. While in the case of sleep habits self-reports methods seem justified, when assessing sleep quality one should go beyond self-reports.
4) In the future, it would also be worth ensuring a larger and more representative group of participants.
5) In conclusion, the authors state that they provided evidence that „chronotype and trait self-control distinctively predicted sleep quality in Chinese adult in Hong Kong”. In my opinion self – control and chronotype are related features and therefore cannot be considered as „distinctively predicted sleep quality”. In addition, the results of the study of a non-representative group of 224 participants (predominantly women, 83.5%) do not provide a basis for generalizing conclusions about the influence of chronotype and self-control on sleep quality in „Chinese adult in Hong Kong ”.

The authors analyze the socially important, extremely current problem of low-quality sleep and try to explain the causes of sleep problems by pointing to the role of features such as chronotype and self-control in regulating sleep-related behavior. The authors also try to point out the mechanism of the influence of these features related to sleep hygiene and bedtime media use. In my opinion, the authors only partially achieved the intended goal, for the reasons indicated above. On the positive side of the work, I would include the analysis of several different sleep hygiene behaviors and the construction of their own method - the Bedtime media use analysis indicator.
