# Supplementary material for: Chronotype and trait self-control as unique predictors of sleep quality in Chinese adults: The mediating effects of sleep hygiene habits and bedtime media use
Source: PLoS One. 2022 Apr 15;17(4):e0266874. doi: 10.1371/journal.pone.0266874 (PMC9012385; doi:10.1371/journal.pone.0266874)
Supplement: Attachment — Submitted filename: Response Letter.docx [file pone.0266874.s005.docx]

Response Letter

Dear Prof. Sirois and reviewers,

We would like to thank you for considering our manuscript for publication in PLOS ONE. We highly appreciate the time and effort you dedicated to providing insightful comments and suggestions to our manuscript. We are grateful for the reviewers’ remarks and constructive feedbacks, as they have helped us tremendously in improving the manuscript.

We have addressed each issue raised by the reviewers, and we believe that the revised manuscript has been improved substantially. Below please find our point-by-point response to the reviewers’ comments.

# **Reviewer #1**

1. Adequacy of Literature Review
a) This manuscript includes much of the relevant literature on the topic, I found it easy to follow and read.
b) Given the now highly notable failures to replicate, I don’t believe the Strength Model of self-regulation is an appropriate theoretical framing of these findings. That being, I don’t believe this theory actually leads to any specific predictions in this paper and I would recommend another dominant model of self-regulation to situate these predictions.

**Response**: Following your suggestion, we have removed the strength model of self-control and relevant arguments. We have included literature review regarding the adaptive function of habits, which we believe better explain our findings (p. 4, lines 69 –80).

2. Theory testing
a) Outside of the issues with using the strength model of self-regulation, I thought the predictions were clearly written and fairly intuitive to follow.
b) The authors state “The relevance of chronotype for sleep hygiene is apparent” but I think this point is not clearly apparent and I would prefer to see a bit more elaboration on how/why there is a link between hygiene and chronotype. Overall, I thought the manuscript was well-written, clearly situated within the literature, and on an important topic that we stand to gain much by understanding further.

**Response**: Thank you for raising this point. We have further elaborated on the link between chronotype and sleep hygiene (pp. 6-7, lines 119–140).

3. Methodology
a) The biggest issue I had with this manuscript is its primary reliance on using cross-sectional, correlational data to analyze the multiple mediation model. I think for this project to represent a meaningful contribution to the literature there needs to be a second study (ideally some kind of experimental work or, perhaps more practically, a longitudinal study, or a case-control approach) with some other, stronger form of evidence. As it stands now, these analyses merely represent a series of concurrent correlational associations already found in the literature. Without stronger forms of evidence, I think the ultimate contribution of this paper is limited. Alternatively, a pre-registered replication might alleviate at least the concern of this pattern of results’ stability.

**Response**: Thank you for your suggestions. We agree that our reliance on cross-sectional and correlational data limit the contributions of this study to the literature. As has been pointed out in the original manuscript, one of the limitations of this study lies in its correlational nature. We agree that stronger forms of evidence, such as longitudinal design and pre-registered replications of the findings, are needed. We have included a paragraph about study design and elaborated more on the need for pre-registered replications of the findings with more rigorous methods in future studies (pp. 30-31, lines 579–588).

b) Were there any exclusion criteria? For example, individuals working the night shift.

**Response**: Thank you for raising this point. Participants who missed three attentional checks or who are shift workers were excluded from the main analyses. We have added the exclusion criteria in the Methods (p. 10, lines 201–204).

4. Statistical Analyses
a) I think, generally speaking, the analyses are well-supported by the literature review.
b) The proposed multiple mediation model section (p. 7, lines 139 – 145) should be placed in its own section following the methods section, prior to the results. Something akin to an Analytic Plan, containing other relevant analytic details to orient readers to the results section.

**Response**: Thank you for your suggestions. We have placed the proposed multiple mediation model in the Analytic Plan section, prior to the results. The relevant analytic details have been added in the Analytic Plan (p. 15).

c) Why were the number of sleep hygiene components derived from an exploratory factor analysis and not from prior psychometric work on the Sleep Hygiene Index.

**Response**: Thank you for raising this point. To our knowledge, there was only one study that examined psychometric properties of the Sleep Hygiene Index in Chinese samples (Tang et al., 2021). We refrained from using the factor structure obtained in that study due to two reasons. First, the sample in Tang et al. (2021) consists of nursing students in mainland China rather than the adult population in Hong Kong. In addition, Tang et al. found a 6-factor model, which did not replicate the 4-factor structure found in past research on psychometric properties of SHI conducted in other countries (e.g., Saudi Arabic; Anwer et al., 2019). Given the inconsistent results with respect to the factor structure of SHI in the literature, we considered conducting exploratory factor analysis to derive the factor structure as more appropriate.

d) Why was age discretized at age 40? It seems more advantageous to leave this in your models as continuous (p. 15, line 287)

**Response**: Thank you for raising this point. We agree that it would be more advantageous to treat age as continuous. However, the participants of this study were instructed to indicate their age group instead of reporting their exact age during the study, as we previously found that the instruction to report the exact age resulted in more response refusal. We now created two dummy variables that better capture the differences between age groups (“21-30 years vs. 31-40 years” and “21-30 years vs. 51 years or above”) and reran the hierarchical regression and mediation analyses (pp. 19-22). The major findings remained unchanged and unaffected by different age coding schemes. To further articulate the measurement of demographic variables, we have added a paragraph that describes control variables in the Measures (p. 14, lines 286– 293).

e) Were the results substantially different when the covariates were omitted from the model?

**Response**: Thank you for your comment. The mediation effects were slightly larger but the main findings remained unchanged when the covariates were omitted from the model. We have added a footnote about this in the revised manuscript (Footnote 2, p. 22).

6. Interpretation

a) I thought the interpretation of the results were clear and well-situated within the larger literature in the discussion section.
b) I would like to see a more involved discussion of the univariate and multivariate descriptive statistics in this sample. I think readers don’t get a very good sense of who this sample really is – are they a more evening oriented group? Is there self-control better than average? As it stands now, the descriptive/preliminary analyses (see p. 13, lines 255 – 264) skews heavily towards describing the sleep behaviors and other media activities, more could be gleaned from other descriptives.

**Response**: Thank you for your suggestion. We have included the descriptive statistics and the distributions of chronotype and trait self-control to give an overview of circadian typology. Additionally, we examined the mean scores in pre-sleep habits and sleep quality between circadian typology groups divided based on Adan and Almirall’s (1991) criterion (pp. 17-18, lines 334 –361).

7. Limitations

a) With 83.5% of the sample comprising women, I wonder the extent to which this sample can reasonably generalized to men.

**Response**: Thank you for this comment, also shared by the other reviewer. We acknowledge that the skewed age- and gender-ratio of our data limit the generalizability of the findings. We have elaborated on this as a limitation in the Discussion section (p. 31, lines 588– 590).

b) Were moderation analyses conducted instead of simply looking at covariates?

**Response**: Thank you for your suggestion. We have run additional analyses to examine whether chronotype or trait self-control moderated the proposed mediation paths (see the Additional Analyses: Moderated Mediation section; pp. 24-25, lines 445–471). We discussed the important findings in the Discussion section (pp. 28-29, lines 533–547).

c) The authors observed fairly low reliability for the rMEQ—I wonder if they could speculate about why this might be the case, as well as if there are any potential solutions for improving reported reliability (e.g. is there an item that seems to be acting up in this sample?).

**Response**: Thank you for your point. The rMEQ has been shown to have an acceptable but not excellent reliability (Cronbach’s alpha is about .6 -.7; e.g., Danielsson et al., 2019). Despite its low reliability, prior work has validated its discriminating power and external validity using circadian motor activity recorded through actigraphy, finding that evening types showed more delayed circadian pattern of motor activity (Natale et al., 2006). Additionally, the rMEQ has been shown to be highly correlated to the full MEQ scale (Milia et al., 2013). We have included a footnote to address the relatively low reliability of the rMEQ scale (see Footnote 1; p. 12).

8. Future directions/applications

a) I think it would be nice to see a richer discussion of how these results might directly facilitate intervention development.
b) Specifically, other work on bedtime procrastination and self-regulation interventions (e.g. Valshtein et al., 2020) and other interventions could be integrated into the final discussion paragraph.

**Response**: Thank you for your suggestion. We have added a section that discusses self-regulation interventions and other implications for sleep hygiene recommendations (pp. 28-29, lines 548– 575).

# Reviewer #2

The authors analyze the influence of chronotype and differences in self-control on the subjective assessment of sleep quality. They assume that the relationship between chronotype, self-control, and sleep quality is mediated by sleep hygiene practices and the bedtime media use. Below, I make comments and remarks on the following parts of the manuscript.
Abstract

a) The statement (p. 2) "Chronotype and trait self-control positively predicted sleep quality" is imprecise and its correct interpretation requires the knowledge that the higher the score on the chronotype measuring scale, the more morning type the person is. The wording "late chronotype" is likewise imprecise.

**Response**: Thank you for pointing this out. We have revised the abstract accordingly. We also removed the wording “late chronotype”, and used “eveningness” throughout the manuscript.

1. Introduction
a) It is not clear to me why the authors start from the self-control strength model and then refer to this model several times since they analyze individual differences in self-control? After all, they do not analyze self-control as a state, but as a trait.

**Response**: Thank you for your important remark, shared with the editor and the other reviewer. We agree with the criticism that the self-control strength model was inappropriate for predicting the findings related to trait self-control. We have removed the model and focused on the adaptive function of habits instead (see p. 4, lines 69 –80).

b) The authors wrote that “their study focuses on two individual differences that determine self-regulation abilities - i.e. chronotype and trait self-control ” (p.3). This sentence requires clarification, in what sense does the chronotype determine the ability to self-regulate? The authors mean the general ability to self-regulate, or the ability to self-regulate sleep behavior?

**Response**: Thank you for raising this point. We agree that a clarification about which type of self-regulation abilities was required. In the revised manuscript, we focused on individual-difference characteristics associated with adaptive habits (see p. 3, lines 43– 46), and we further elaborated on the mechanisms underlying low capacities to regulate pre-sleep behaviors in evening types (see pp. 6-7, lines 119– 140).

c) Again, the authors describe the self-control strength model and then state that the self-control trait is a stable individual difference (p.3). In the self-control strength model, intra-individual differences in self-control are analyzed, not differences between individuals.

**Response**: Thank you for your careful reading. We have removed all the statements related to the self-control strength model.

d) The authors repeated that "another characteristic that determines self -regulation abilities and contributes to sleep quality is chronotype." (p.5) Again, are the authors concerned with general self-regulation abilities or just regulation of sleep behavior?

**Response**: Thank you for your comment. In the revised manuscript, we now stated that chronotype is another individual difference that we hypothesized would determine the ability to regulate pre-sleep behaviors (see p. 6, lines 109-111).

e) In the part describing the relationship between the chronotype and sleep hygiene and sleep quality, the authors mention the relationship between eveningness and procrastination, often confirmed in research (p. 6). However, the authors do not mention that the research also indicates a relationship between chronotype and self-control trait. Thus on the basis of the research conducted so far, the assumption made by the authors about the independent effect of self-control and chronotype seems unjustified. While it is still unclear about the direction of the relationship between self-control and chronotype, it seems obvious that the two features are related. It also means that the model of analysis adopted by the authors in the following part is not justified and raises significant doubts.

**Response**: Thank you for your important remark. We have included relevant literature to strengthen the link between chronotype and trait self-control (see p. 7, lines 141–153).

2. Statistical analysis
a) Why do the authors provide age ranges, but mean value, standard deviation, and age range are missing? Why do the authors divide age into two categories under 40 and over 41 instead of including age as a continuous variable? Age is an important factor influencing the chronotype and quality of sleep. With this approach to this variable, the relationship between age and chronotype and sleep quality is impossible to capture.

**Response**: Thank you for raising this point, also shared by the editor and the other reviewer. We agree that it is important to assess the relationship between age, chronotype and sleep quality. However, age was measured as a categorical variable in our study. Please find more details in Response to Reviewer 1’s comment 4d.

b) The results of the correlation analysis confirm that self-control and chronotype are significantly (p <.001) related. In my opinion, this relationship does not allow these variables to be included in the analysis as two distinctive independent variables.

**Response**: Thank you for your comment. Despite the correlation between trait self-control and chronotype, the results of multiple regression analyses found that chronotype contributed *additionally* to explain the variance in sleep quality when trait self-control was entered as an independent variable. and vice versa. As Cohen (1968) pointed out that the b_i_ refers to “*the multiple-partial correlations with the criterion, that is, the correlation of Y with X_i_, partialing out or holding constant all the other independent variables*” (p. 431; see also Cohen et al., 2002). If trait self-control and chronotype do not represent two distinctive/independent predictors of sleep quality, we would have found a nonsignificant effect of chronotype when trait self-control was entered in the model. This was apparently not the case, which further supports our proposition that the two predictors have their unique contributions to sleep hygiene behaviors and sleep quality.

c) Further analyzes confirm that the effect of self-control on sleep quality is bigger than that of the chronotype and that it is both direct and indirect.

**Response**: Thank you for your careful reading. We have noted this difference in the Result section (see p. 26, lines 481–486).

3. Discussion
a) The authors wrote: “In accordance with the self-control strength model, the abilities to downregulate stress and emotion (e.g. self-regulatory abilities) underlie the differences in sleep quality between morning and evening types and between people high vs. low in trait self control”. (p.20) - I do not understand in what sense it is consistent with the self-control strength model? In addition, it is about abilities or some habitual behaviors? Sleep hygiene is associated with certain healthy habits, not abilities.

**Response**: Thank you for your suggestions. We have removed the statement related to the self-control strength model and revised the sentence (see pp. 26-27, lines 496–498).

b) The discussion does not explain the lack of influence on sleep quality in the studied group of such sleep hygiene behaviors as sleep timing regularity and avoiding sleep-disrupting food or activities. Why did these behaviors, known to positively affect sleep quality, not have such an impact on sleep quality in the study group?

**Response**: Thank you for your suggestion. Multiple mediation approach allows several mediators to compete against each other for the variance in sleep quality (Preacher & Hayes, 2008). The absence of mediation effects of sleep timing regularity and avoiding sleep-disrupting food or activities suggests that indirect effects associated with these two components were largely reduced in the presence of the other prevailing mediators that accounted for the associations between chronotype, trait self-control and sleep quality. To address your concern, we have included the purpose of multiple mediation model in the Introduction (see pp. 8-9, lines 173–183), as well as its interpretations for the relative magnitude of multiple mediators in the Discussion section (see pp. 25-26, lines 477-479 and lines 499–508).

c) Pointing to the limitations of the conducted study, the authors emphasize that sleep habits should be measured in a different way in the future. While in the case of sleep habits self-reports methods seem justified, when assessing sleep quality one should go beyond self-reports.

**Response**: Thank you for your suggestion. We have further elaborated more on the self-report method of sleep quality as a major limitation in the Discussion section (see p. 31, lines 590– 595).

d) In the future, it would also be worth ensuring a larger and more representative group of participants.

**Response**: Thank you for your suggestion. We have added a statement regarding this in the Discussion section (see p. 31, lines 588–590), and we will keep this in mind for planning future studies.

e) In conclusion, the authors state that they provided evidence that “chronotype and trait self-control distinctively predicted sleep quality in Chinese adult in Hong Kong”. In my opinion self–control and chronotype are related features and therefore cannot be considered as “distinctively predicted sleep quality”. In addition, the results of the study of a non-representative group of 224 participants (predominantly women, 83.5%) do not provide a basis for generalizing conclusions about the influence of chronotype and self-control on sleep quality in “Chinese adult in Hong Kong ”.

**Response**: Thank you for your comment. Please find our arguments with respect to the distinctive roles of trait self-control and chronotype in Response to Reviewer 2’s Comment 2b. Although a meta-analytic study reported that the proportion of females in the sample did not moderate the strength of the relationship between self-control and sleep quality (Guarana et al., 2021), we agree that convenient sampling of this study, as well as the skewed gender ratio limit the generalizability of our findings to the entre Chinese adult population in Hong Kong. We have pointed this out as a major limitation (see p. 31, lines 588– 590).

The authors analyze the socially important, extremely current problem of low-quality sleep and try to explain the causes of sleep problems by pointing to the role of features such as chronotype and self-control in regulating sleep-related behavior. The authors also try to point out the mechanism of the influence of these features related to sleep hygiene and bedtime media use. In my opinion, the authors only partially achieved the intended goal, for the reasons indicated above. On the positive side of the work, I would include the analysis of several different sleep hygiene behaviors and the construction of their own method - the Bedtime media use analysis indicator.

**Response**: Thank you for your constructive comments and appreciation of our work. We have addressed each issue you raised (as listed above), and we hope that you find the revised manuscript satisfactory.

References

Anwer, S., Alghadir, A., Manzar, M. D., Noohu, M. M., Salahuddin, M., & Li, H. (2019). Psychometric Analysis Of The Sleep Hygiene Index And Correlation With Stress And Anxiety Among Saudi University Students. *Nature and Science of Sleep*, *11*, 325-332. https://10.2147/NSS.S222440

Cohen, J. (1968). Multiple regression as a general data-analytic system. *Psychological Bulletin*, *70*(6, Pt.1), 426-443. https://10.1037/h0026714

Cohen, J., Cohen, P., West, S.G., & Aiken, L.S. (2002). *Applied Multiple Regression/Correlation Analysis for the Behavioral Sciences* (3rd ed.). Routledge. https://doi.org/10.4324/9780203774441

Danielsson, K., Sakarya, A., & Jansson-Fröjmark, M. (2019). The reduced Morningness-Eveningness Questionnaire: Psychometric properties and related factors in a young Swedish population. *Chronobiology International*, *36*(4), 530-540. https://10.1080/07420528.2018.1564322

Guarana, C. L., Ryu, J. W., O'Boyle, E. H., Lee, J., & Barnes, C. M. (2021). Sleep and self-control: A systematic review and meta-analysis. *Sleep Medicine Reviews*, *59,* 101514. https://10.1016/j.smrv.2021.101514

Milia, L. D., Adan, A., Natale, V., & Randler, C. (2013). Reviewing the Psychometric Properties of Contemporary Circadian Typology Measures. *Chronobiology International*, *30*(10), 1261-1271. https://10.3109/07420528.2013.817415

Natale, V., Esposito, M. J., Martoni, M., & Fabbri, M. (2006). Validity of the reduced version of the Morningness-Eveningness Questionnaire. *Sleep and Biological Rhythms*, *4*(1), 72-74. https://10.1111/j.1479-8425.2006.00192.x

Preacher, K. J., & Hayes, A. F. (2008). Asymptotic and resampling strategies for assessing and comparing indirect effects in multiple mediator models. *Behavior Research Methods*, *40*(3), 879-891. https://10.3758/BRM.40.3.879

Tang, Z., Li, X., Zhang, Y., Li, X., Zhang, X., Hu, M., & Wang, J. (2021). Psychometric analysis of a Chinese version of the Sleep Hygiene Index in nursing students in China: a cross-sectional study. *Sleep Medicine*, *81*, 253-260. https://10.1016/j.sleep.2021.02.050
